# Evaluation of Grain Boundary Network and Improvement of Intergranular Cracking Resistance in 316L Stainless Steel after Grain Boundary Engineering

**DOI:** 10.3390/ma12020242

**Published:** 2019-01-12

**Authors:** Tingguang Liu, Shuang Xia, Qin Bai, Bangxin Zhou, Yonghao Lu, Tetsuo Shoji

**Affiliations:** 1 National Center for Materials Service Safety, University of Science and Technology Beijing, Room 622, Tuhuan Building, No. 30 Xueyuan Road, Haidian District, Beijing 100083, China; lu_yonghao@mater.ustb.edu.cn (Y.L.); tshoji@fri.niche.tohoku.ac.jp (T.S.); 2 School of Materials Science and Engineering, Shanghai University, Shanghai 200072, China; xs@shu.edu.cn (S.X.); baiqin31@shu.edu.cn (Q.B.); zhoubx@shu.edu.cn (B.Z.); 3 Frontier Research Initiative, NICHe, Tohoku University, Sendai 980-8579, Japan

**Keywords:** austenitic stainless steel, EBSD, grain boundary engineering, grain boundary connectivity, stress corrosion cracking

## Abstract

For understanding the improvement of intergranular stress corrosion cracking (IGSCC) propagation in grain boundary engineering (GBE)-processed metals exposed to a simulated pressurized water reactor (PWR) environment, characteristics of the grain boundary network of 316L stainless steel before and after GBE were investigated and compared, including proportions both in length and in number of ∑3*^n^* boundaries, sizes, and topology of grain clusters (or twin-related domains), and connectivity of random boundaries. The term through-view random boundary path (TRBP) was proposed to evaluate the random boundary connectivity. A TRBP is a chain of end-to-end connected crack-susceptible boundaries that passes through the entire mapped microstructure. The work provides the following key findings: (I) the length fraction of ∑3*^n^* boundaries was increased to approximately 75% after GBE, but the number fraction was only approximately 50%; (II) a connected non-twin boundary network still existed in the GBE sample due to the formation of grain clusters; (III) the GBE sample exhibited a higher resistance to IGSCC; and (IV) as the twin boundary fraction increased, the number of TRBPs decreased and the normalized length of the minimum TRBP increased monotonically, leading to a higher resistance to IGSCC.

## 1. Introduction

The improvement of resistance to intergranular failure of polycrystalline metallic materials has presented critical industry problems, such as intergranular corrosion (IGC) [1,2,3,4] and intergranular stress corrosion cracking (IGSCC) [5,6,7] as well as intergranular segregation/precipitation [8]. These problems are especially evident in austenitic stainless steels and Ni-based alloys exposed to light water reactor environments. The synergistic effects of the corrosive condition and structural stress which are unavoidable due to the high temperature and high pressure water and the engineering structure caused these intergranular failures [9]. Grain boundary engineering (GBE) based on “grain boundary control and design”, which was first proposed by Watanabe in 1984 [10], has been demonstrated as a promising method to mitigate the intergranular degradation according to extensive investigations carried out in the last decades [1,2,3,4,5,6,7]. Instead of modifying the mechanics or chemistry [11], GBE provides a methodology to prevent intergranular degradation by control of the grain boundary (GB) network of materials, based on the idea that the percolation process of intergranular failure could be avoided if the proportion of corrosion-resistant boundaries was high enough in the GB network [1,12].

Two of the early successful applications of GBE for improving GB-related properties of materials were carried out by Lin et al. [13] and Lehockey et al. [14]. Lin et al. showed that the resistance to sensitization and intergranular corrosion of Alloy 600 increased commensurately with the fraction of the so-called special boundaries (low-*∑* coincident site lattice (CSL) boundaries, *∑* ≤ 29) [13]. The work of Lehockey et al. revealed that the service life of positive Pb-acid battery grids after GBE treatment was two- to four-fold longer than the conventional grids [14]. Numerous recent studies [1,2,3,4,5,6,7,15] show similar results. The intergranular corrosion rate decreases as the proportion of low-*∑* CSL boundaries increases.

The proportion of special boundaries had been treated as a critical matter leading to the improved resistance to intergranular degradations in most of the past GBE studies [1,2,3,4,13,14]. It is clearly evident that special boundaries, specifically *∑*3 (the twin boundaries), have a higher resistance to intergranular degradation [5,7,16,17]. However, in the last decade, a growing number of studies [1,12,18,19,20,21] have paid increasing attention to the topological characteristics of the entire GB network, considering that the proportion is not sufficient to evaluate the intergranular properties in terms of GBE. For example, a previous work [7], which was carried out by some of the present authors, Liu et al., showed that an intergranular crack propagated a long distance in GBE-processed 316 stainless steel, although the steel had a high proportion of low-*∑* CSL boundaries (i.e., more than 70%). The connectivity of corrosion-susceptible random GBs represents more informative data because the propagation of intergranular failures is primarily affected by the random boundary network. The intergranular failure growth is arrested if the random boundary connectivity is interrupted by special boundaries. Therefore, disrupting the connectivity of a random boundary network is more important for GBE processing. The high proportion of special boundaries is essential because percolation theory shows that the threshold fraction of special boundaries is approximately 0.85 in order to disrupt the random boundary connectivity [12], but the high proportion does not necessarily result in a disrupted random boundary network. In addition to proportion, the distribution characteristics of special boundaries in a GB network are another important factor in evaluating the connectivity of a GB network [20].

The distributions of CSL boundaries in a GB network are subject to crystallographic constraints at both triple junctions and quadruple junctions [12]. A triple junction has two *∑*3 boundaries at most and a *∑*9 boundary in this case; a quadruple junction has three *∑*3 boundaries at most and two *∑*9 boundaries and a *∑*27 boundary in this case [22]. In addition, the mechanism of GB network evaluation during GBE, that is, the multiple-twinning process [23,24,25,26], gives rise to the formation of grain clusters (or called twin-related domains) [24,25,26,27,28,29] which are assemblies of twins. All GBs within a grain cluster have twin-related misorientations (*∑*3*^n^*-type), and the interfaces between grain clusters are crystallographically random boundaries. Consequently, connected crack-susceptible GBs exist in the materials even after GBE, which creates pathways for IGSCC propagation [7]. On the other hand, it is true that GBE processing increases the resistance to the intergranular fracture of materials [1,3,4,5,6,7,15,30]. For these reasons, improving the resistance of intergranular degradation of materials is still challenging and will be the aim of further studies on how to precisely predict the effectiveness of GBE. 

This study aims to find better methods to evaluate the GB network after GBE processing so that we can predict the resistance of materials to intergranular failures. A Thermo-mechanical processing route was developed to optimizing the GB network in large-size 316L stainless steel in terms of GBE. Stress corrosion cracking (SCC) testing was performed to compare the behaviors of intergranular cracking of the steel before and after GBE. The topological characteristics of grain clusters and random boundary connectivity and their effects on IGSCC propagation were investigated. Furthermore, a quantity, termed through-view random boundary path (TRBP), was proposed to evaluate the connectivity of random boundaries.

## 2. Experimental Methods

### 2.1. Materials

The chemical composition of 316L stainless steel used in this study is given in Table 1. A block with dimensions of 40 × 130 × 1190 mm^3^ was fabricated using wire-cut electrical discharge machining from the as-received forged 316L plate. The block was hot rolled by a 50% thickness reduction at a starting temperature of 950 °C followed by an immediate water quench. A part of the hot rolled steel was solution annealed at 1100 °C for 90 min and water quenched, obtaining the conventional sample (non-GBE) of this work. To obtain the GBE sample, the other part of the hot rolled steel was annealed at 950 °C for 30 min followed by thermo-mechanical processing (TMP) according to our previous studies [7,31]: warm rolling with one pass to a 5% thickness reduction at a starting temperature of 400 °C followed by annealing at 1100 °C for 60 min and water quenching. The GBE processing route, which is warm rolling with a low strain deformation followed by a solution annealing, was particularly developed to process large-size materials, because the warm rolling produces a relatively uniform deformed microstructure on the cross-section. The final thickness of the as-prepared GBE sample was 19 mm.

**Figure 1:**
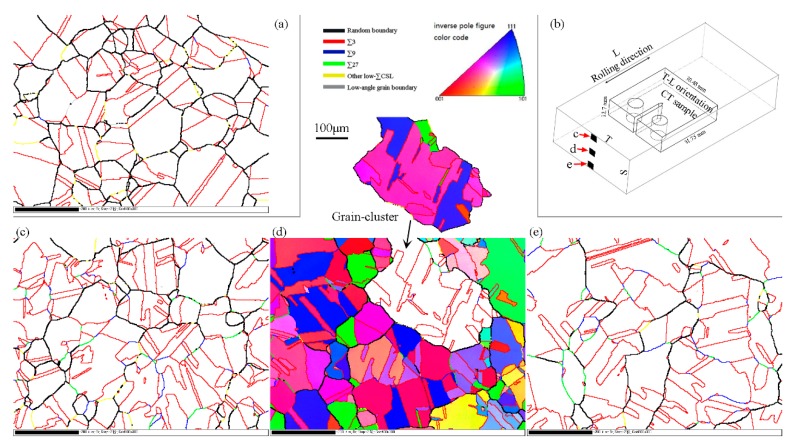
(**a**) Electron backscatter diffraction (EBSD) map of 316L stainless steel before grain boundary engineering (GBE) processing. (**b**) Schematic showing the orientation of CT specimen and positions of EBSD mapping on the cross-section of GBE sample. (**c**–**e**) EBSD maps collected on the GBE 316L stainless steel, of which the positions are corresponding to the labels on (**b**). A grain cluster was highlighted from the middle EBSD map. The scale bars for (**a**,**c**–**e**) are 200 μm.

An Oxford Instruments/HKL Electron Backscatter Diffraction (EBSD) system (Oxford, UK), which was linked to a CamScan Apollo 300 Scanning Electron Microscope (SEM) (Cambridge, UK), was used to characterize the CSL boundaries. For EBSD mapping, the sample surface was ground using waterproof silicon carbide papers from 160 grit to 2400 grit followed by a final electro-polishing in an electrolyte 20% HClO_4_ + 80% CH_3_COOH at room temperature using 30 V direct current for 90 s. The final electro-polishing can remove the surface deformation caused by the prior mechanical grinding. Three positions on the cross-section of the GBE sample, located at the upper, middle, and bottom parts, respectively, were mapped using the EBSD system with a step size of 2 μm, as shown in Figure 1. The CSL boundaries were defined according to the Brandon criterion [32].

### 2.2. Stress Corrosion Cracking

First, the as-prepared non-GBE and GBE 316L stainless steel samples were sensitized at 650 °C for 12 h. Subsequently, compact tension (CT) specimens were machined by a wire-cut electrical discharge machining from the two sensitized samples. The specimens were 1/2T CT (thickness B = 12.5 mm) with T-L orientation according to ASTM E399, where “L” means longitudinal direction (rolling direction), and “T” means long transverse direction, as shown in Figure 1. 

Before the SCC test (Slow strain rate testing machine system, Toshin Kogyo Co., Ltd., Tokyo, Japan), the CT specimens were first pre-cracked by fatigue under a sine-wave loading in air with a frequency of 20 Hz, a load ratio of *R* = 0.2, and a maximum stress intensity factor of *K*_max_ = 20.7 MPa√m at the starting stage and *K*_max_ = 13.8 MPa√m at the ending stage. As a result, the length of the pre-crack was 1.71 mm for the non-GBE CT sample and 1.76 mm for the GBE CT sample, respectively. Subsequently, the two CT specimens were fixed in series in an autoclave that was linked with a slow strain rate tensile (SSRT) system for the SCC test; therefore, the SCC performances of the two samples were compared under the same load and water conditions. The testing condition within the autoclave was simulated pressurized water reactor (PWR) primary water: 1200 ppm (in mass) B^3+^ (as H_3_BO_3_), 2 ppm (in mass) Li^+^ (as LiOH), dissolved oxygen concentration (DO) at 8.0 ppm, temperature at 320 °C, and pressure at 13.0 MPa. A transition procedure, using a fatigue loading of a triangular-wave form at a load ratio of *R* = 0.3, 0.5, and 0.7 for 8 h, 16 h, and 24 h, respectively, was carried out before SCC testing. The applied *K*_max_ was approximately 21.6 MPa√m for both samples, and the frequency was 0.01 Hz during the whole transition stage. The transition stage was performed for propagating the crack through the previous plastic zone produced by fatigue pre-cracking in air, promoting the transition of the crack propagation from a transgranular form to an intergranular form. After the transition stage, the specimens were subjected to a constant loading (4.98 kN) (i.e., the SCC testing period). The calculated *K* applied to both samples was approximately 21.6 MPa√m at the SCC starting stage. The constant loading period was 1640 h.

The SCC-tested samples were cut evenly in half by using an electro-discharge machine. They were used to reveal the fractography and profile microstructure, respectively. SEM, optical microscopy (OM), and EBSD were used to investigate the SCC behaviors of the two samples. Post-cracking by fatigue in air was carried out on the half samples for fracture surface observation. The other half samples were ground using waterproof silicon carbide papers from 160 grit to 2400 grit followed by a final mechanical polishing using a Buehler 40-7920 ChemoMet synthetic polishing cloth and 40-6377 MasterPrep polishing suspension (0.05 μm alumina), so that the profile morphology of the SCC propagation path could be mapped by OM and EBSD. The final mechanical polish removed the surface deformation caused by the prior mechanical grinding, which resulted in a good surface for EBSD collection.

## 3. Results

### 3.1. Grain Boundary Character Distribution (GBCD)

The microstructures, which were measured using EBSD, of the conventional (non-GBE) 316L stainless steel and of the same material after GBE processing are shown in Figure 1. One position (Figure 1a) was measured on the middle of the non-GBE sample’s cross-section, considering it should have a uniform microstructure. Three positions (Figure 1c–e) were measured on the GBE sample’s cross-section to check the uniformity of the GBE microstructure. The positions are shown in Figure 1b.

**Figure 2:**
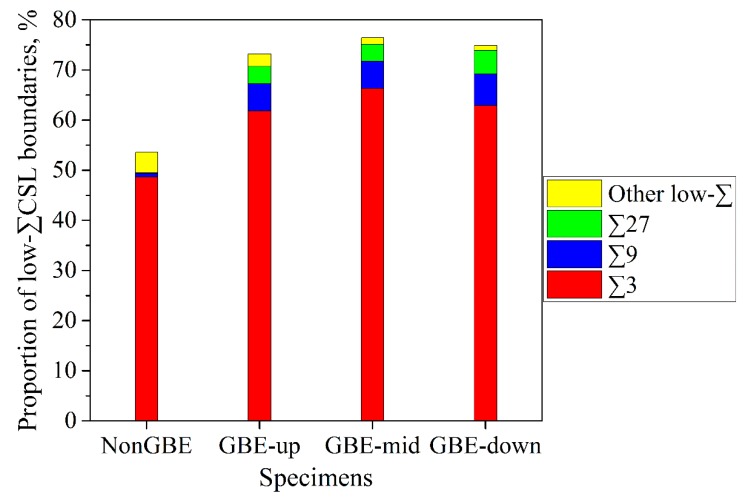
Grain boundary character distributions (GBCDs) in the four EBSD maps, corresponding to Figure 1, of the non-GBE and GBE 316L stainless steel samples.

The EBSD maps in Figure 1 demonstrate that the GBE sample has a higher fraction of low-*∑* CSL boundaries than the non-GBE sample as obtained by past studies [21,33,34,35,36]. The fractions of low-*∑* CSL boundaries are 73.2%, 76.4%, and 74.9% for the upper, middle, and lower positions in the GBE sample, respectively, as shown in Figure 2. The average fraction of low-*∑* CSL boundaries in the three maps of the GBE sample is 74.8% (boundary length fraction), and the average fraction is 53.6% for the non-GBE sample. In comparison, the three positions in the GBE sample have a similar microstructure with a similar high fraction of special boundaries, suggesting that the GBE sample has a uniform microstructure through the cross-section, although the thickness of the sample is quite large (19 mm). The process of warm rolling with a low strain deformation plus annealing is a favorable thermomechanical procedure for large-size samples in terms of GBE.

**Figure 3:**
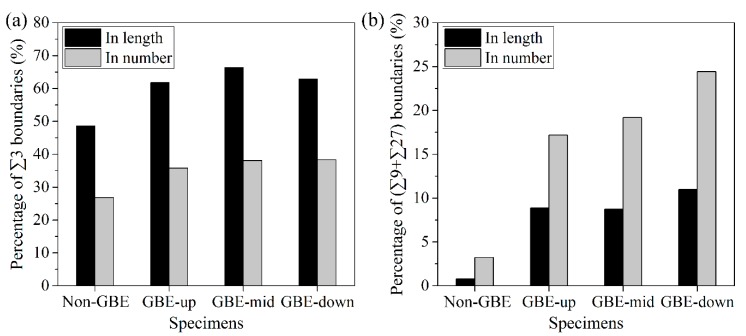
Length fractions and number fractions of (**a**) twin boundaries (*∑*3) and (**b**) *∑*9 plus *∑*27 boundaries in the non-GBE and GBE samples.

In addition, Figure 2 shows that more than 80% of the low-*∑* CSL boundaries are twin boundaries (*∑*3), and more than 90% of them are twin-related boundaries (*∑*3*^n^*). This result is caused by the multiple-twinning process [23,24,25,26] that occurs during the GBE processing’s annealing stage. This will be discussed in the next section. Although Figure 2 shows a high fraction of CSL boundaries in the GBE 316L, it should be noted that these data were calculated using GB lengths rather than numbers. The number fractions of CSL boundaries are quite different from the length fraction, as shown in Figure 3. In comparison, the number percentages of twin boundaries are generally lower by 20 to 30 percentage points than their length percentages, but the number percent of *∑*9 plus *∑*27 boundaries is generally higher by 2 to 16 percentage points than the length percent. This is in agreement with Kumar et al. [37], Randle and Coleman [33,38], and a three-dimensional study by the present authors [39]. Additionally, the difference between the number and length fractions of the GBE sample is larger than the difference for the non-GBE sample. The phenomenon is also correlated with the multiple-twinning process as discussed in the next section.

### 3.2. Topological Characteristics of Grain clusters

**Figure 4:**
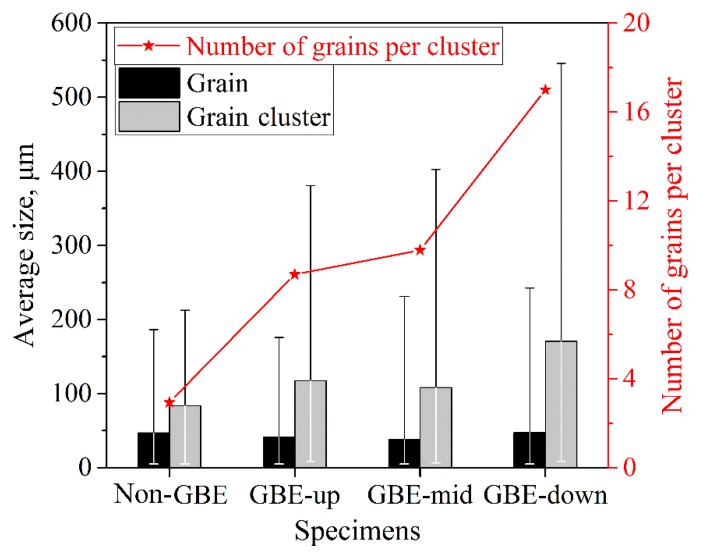
Average grain sizes, average grain cluster sizes, and average numbers of grains per cluster in the non-GBE and GBE samples.

Grain cluster sizes are widely measured when one evaluates the microstructures of GBE materials [24,25,26,27,28,29]. The large size of grain clusters is a prominent feature of GBE-processed microstructures. For example, the size (equivalent circle diameter, the same below) of the grain cluster highlighted in Figure 1d is 335 μm. Figure 4 shows the average sizes of grains and grain clusters in the non-GBE and GBE samples. Although the GBE sample has a slightly smaller average grain size (42.1 μm) than the non-GBE sample (47.0 μm), the GBE sample has much larger grain cluster sizes (132.2 μm on average) than the non-GBE sample (83.4 μm). The grain cluster size distributions are quite non-uniform. Some grain clusters have extremely large sizes. The size of the largest grain cluster in the GBE sample is more than 546 μm, which is beyond the view of EBSD mapping.

A grain cluster is an assembly of twin-related grains which are formed by a multiple-twinning process [23,24,25,26,27]. Twinning operations can occur during the recrystallization of face-centered-cubic materials with low-to-medium stacking fault energy [40,41,42], such as 316L stainless steel. Multiple-twinning is a repeated process of twinning operations starting from a single nucleus during recrystallization. As a result, a sequence of twins will be formed from the nucleus, constructing a grain cluster.

Twinning operations are commonly known as “growth accidents” in traditional studies of recrystallization [40,41,42], because the occurrences of twinning were believed to be a random event. However, annealing twins can resume the growth of stagnant grains during recrystallization with a low driving force according to the literature by Field et al. [43], specifically for the GBE process. A typical thermo-mechanical procedure in terms of GBE is pre-strain by a low deformation plus annealing [35,36,44,45,46], as is the method used in this work. The low strain is not always enough to promote the migration of recrystallized front boundaries during annealing [47]; the twinning operation can then alter the misorientation of migrating front boundaries [43,48]. It is possible that the twinning operation produces a new front boundary with higher mobility, thereby promoting the process of recrystallization [46,48]. Therefore, twinning operations become “necessary incidents” to a large extent to motivate a full recrystallization route during GBE. 

The multiple-twinning occurs not only during GBE processing but also during conventional recrystallization, but the extents of multiple-twinning are different for the two types of processes [27,28,29]. The number of cycles of a multiple-twinning process is approximately equal to the number of twins within the formed grain cluster. In Figure 4, the red line indicates the average number of twins per grain cluster in the four maps in Figure 1. It shows that the grain clusters in the GBE sample embrace more twins than in the non-GBE sample. Therefore, the multiple-twinning processes during GBE perform more cycles than those during conventional recrystallizations. The grain clusters in GBE samples are commonly larger than those in conventional materials, as shown in Figure 4.

**Figure 5:**
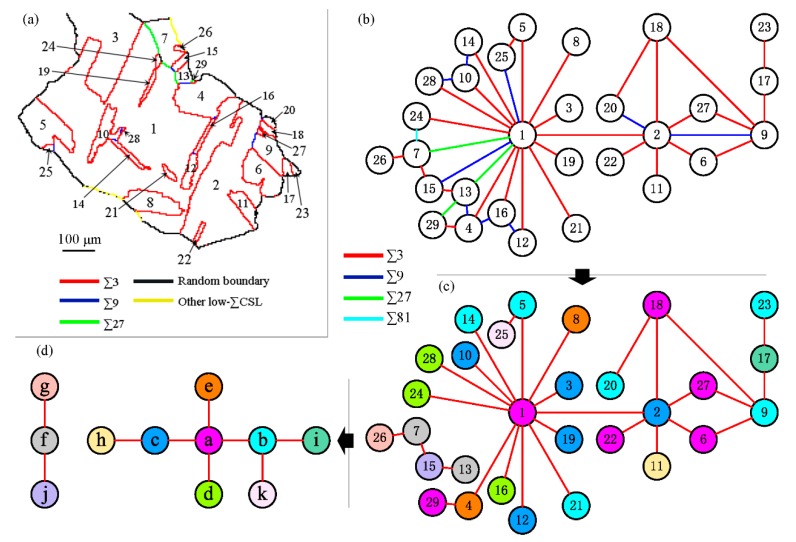
Topological analysis of the highlighted grain cluster in Figure 1d: (**a**) the 29 grains within the grain cluster are labeled using Arabic numerals; (**b**) topological network of the 29 grains, in which the boundary characters were differentiated by colors; (**c**) twin-chain of the 29 grains. The 29 grains belong to 11 orientations, designated as a~k (a: 1, 6, 18, 22, 27, 29; b: 5, 9, 14, 20, 21, 23; c: 2, 3, 10, 12, 19; d: 16, 24, 28; e: 4, 8; f: 7, 13; g: 26; h: 11; i: 17; j: 15; k: 25.). (**d**) Twin-chain of the 11 orientations which are differentiated by using the same color code as (**c**).

As a grain cluster is formed by multiple-twinning, all grains within it can be connected by a twin-chain. For example, Figure 5 shows the twin-chain of the grain cluster highlighted in Figure 1d. The grain cluster has 29 grains which were labeled using Arabic numerals following the order of area from large to small. Figure 5b topologically shows the topology of the GB network between the 29 grains. All the boundaries are *∑*3*^n^*-type in terms of CSL theory. Figure 5c shows the twin-chain between the 29 grains, in which other types of boundaries were omitted but for the twin boundaries according to Figure 5b. Grains 7, 13, 15, and 26 are not connected with the other grains by twin boundaries. This is caused by the two-dimensional (2D) characterization. Apart from the 29 grains, the grain cluster must include other grains, and then the four grains can be connected with the other grains by twin boundaries. This can be confirmed by three-dimensional (3D) characterization [20,27].

The 29 grains belong to 11 orientations. Figure 5d shows the twin-chain of the 11 orientations. It represents the orientation evolution route during the multiple-twinning [24,27,49]. When two grains encounter as grain growth within a grain cluster, they form a *∑*3*^n^*–type GB, where the value *n* is equal to the number of steps between the two orientations that the two grains belong to according to Figure 5d. The frequency of the grain encountering with longer distances in the twin-chain of orientation is lower. Therefore, the percentage of higher-order *∑*3*^n^* boundaries is lower. In the grain cluster in Figure 5a, there are 42 boundaries. The numbers of *∑*3, *∑*9, *∑*27, and *∑*81 boundaries are 29, 9, 3, and 1, respectively. 

According to the multiple-twinning process, the *∑*3*^n^* boundaries within a grain cluster have two formation modes, namely, twinning operation and grain encounter. All the high-order *∑*3*^n^* boundaries were formed by the grain encounter mode. Twining operations definitely forms twin boundaries. Thus, most of the twin boundaries within a grain cluster are formed by the twinning operations, but the grain growths and encounters may form twin boundaries as well.

The difference between the number fraction and length fraction of the *∑*3*^n^* boundaries, as shown in Figure 3, can be correlated with the formation modes. The length fractions of high-order *∑*3*^n^* boundaries are generally lower than their number fractions, indicating that the high-order *∑*3*^n^* boundaries that were formed by grain encounters during multiple-twinning are generally smaller than other types of GBs on average. Conversely, the twin boundaries, most of which were formed by twinning operations, have higher length fractions than number fractions, suggesting that the twin boundaries are generally larger than the other types of GBs that were formed by grain encounters.

In addition, according to the multiple-twinning process, the twin-chain of grain cluster is a tree-shape structure [27,49]. However, circle structures were observed in the twin-chain of Figure 5c, such as 2-6-9-27-2. This feature is correlated with grain encounters. In each circle structure of a twin-chain, there must be a twin boundary that was formed by a grain encounter, or there is a grain that was formed by the encounter of two grains with the same orientation. For example, a possible route to form the circle 2-6-9-27-2 is 2→6→9→27, where ‘→’ indicates a twinning operation. Grains 2 and 27 encountered as they were growing. They have twin-related misorientation, so a twin boundary formed when they encountered. Figure 5a shows that the twin boundary between grains 2 and 27 is quite small. Additionally, Figure 5c shows only a few circle chains, indicating that only a few twin boundaries within the grain cluster were formed by grain encounters. Most of the twin boundaries were formed by twinning operations. The frequency to form a circle chain is quite low because the circle chain requires an evolutionary history of at least three twinning operations plus one grain encounter. The occurrence of such an evolutionary route is a matter of chance rather than choice.

### 3.3. Evaluation of Random Boundary Network

**Figure 6:**
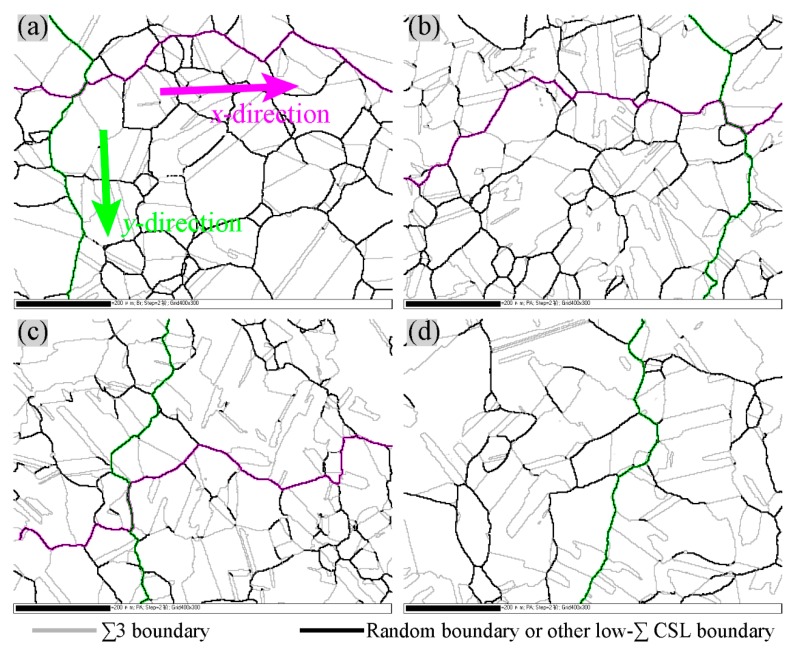
Connectivity of non-twin boundaries (random boundaries and low-*∑* CSL boundaries except *∑*3) of the (**a**) non-GBE, (**b**) GBE-up, (**c**) GBE-mid, and (**d**) GBE-down, which correspond to Figure 1. All scale bars of the four maps are 200 μm.

As described above, the formation of grain clusters during GBE processing necessarily results in connected random boundaries regardless of the level of special boundary proportion [7,27,28]. In particular, when all non-twin boundaries were considered as failure-susceptible boundaries [3,4,7], a connected network of failure-susceptible boundaries was retained more easily after GBE. As examples shown in Figure 6, both the 316L stainless steel samples before and after GBE have connected non-twin boundaries, although the length fraction of the twin boundaries is quite high for the GBE sample (approximately 65%). On the other hand, while the four microstructures have a long-range connected random boundary network (non-twin boundaries), the frequency and minimum length of the random boundary paths through the entire view of these microstructures may be different. Therefore, the term through-view random boundary path (TRBP) was proposed in this work. A TRBP is a chain of end-to-end connected crack-susceptible boundaries, which passes through the entire mapped microstructure from left to right (in the *x*-direction) or from top to bottom (in the *y*-direction).

The TRBP indicates a pathway along which the intergranular degradation can propagate through the entire EBSD map, such as the pink highlighted pathways in Figure 6a. In this case, if the EBSD mapping field of view represents the whole microstructure of the specimen, the intergranular failures propagate through the specimen along the TRBPs, thus breaking the specimen. The map of the non-GBE sample (Figure 6a) has many TRBPs. In comparison, the numbers of TRBPs in Figure 6b–d (GBE sample) are less, and there is even no TRBP in the *x*-direction in Figure 6d. Therefore, GBE materials with a higher fraction of twin boundaries tend to have a fewer number of TRBPs.

The minimum TRBP of a microstructure is the most feasible pathway that an intergranular failure propagates through the sample, because it is the shortest pathway. Thus, the length of the minimum TRBP has a tight relationship with the resistance of materials to intergranular failure. The minimum TRBPs in the four microstructures in the *x*-direction and *y*-direction are highlighted in pink in Figure 6, noting that the minimum paths were identified artificially. For a certain size of microstructures, a longer minimum TRBP indicates that the intergranular failure has to propagate through a more winding pathway, taking a more zigzag route and making propagation difficult. Therefore, a normalized length (*D*_R_) is given by the ratio of the length of the minimum TRBP in the *x*-direction (*L*_TRBP-X_) or *y*-direction (*L*_TRBP-Y_) to the width (*X*) or height (*Y*) of the view of a measured microstructure as follows:

(1) $$D_{R-X}=\frac{L_{\mathrm{TRBP}-X}}{X},\;D_{R-Y}=\frac{L_{\mathrm{TRBP}-Y}}{Y}.$$

**Figure 7:**
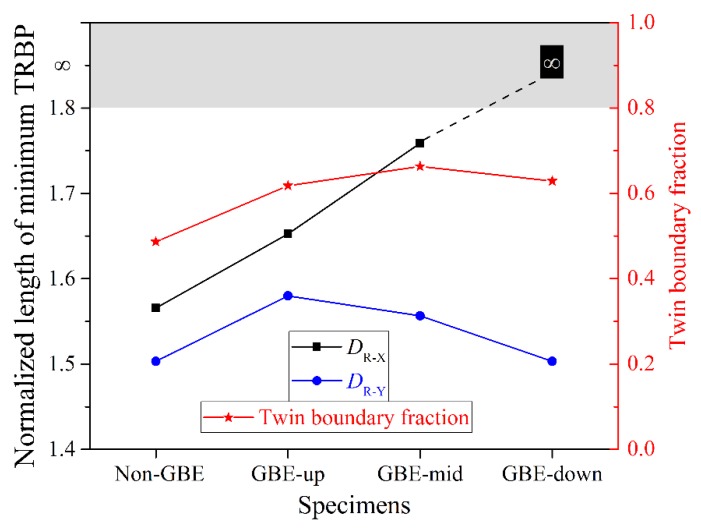
Statistics of the minimum through-view random boundary path (TRBP) and twin boundary fractions in the four maps in Figure 6.

The *D*_R_-values of the four microstructures in Figure 6 are presented in Figure 7. The lengths of the minimum TRBPs were measured using ImageJ (v1.51s) [50]. A microstructure with a higher fraction of twin boundaries tends to have a larger *D*_R_ (a further discussion is found in Section 4.2), but there is a difference between *D*_R-X_ and *D*_R-Y_. The values of *D*_R-X_ of the GBE sample are obviously larger than that of the non-GBE sample. The map of GBE-down has no TRBP in the *x*-direction, suggesting that intergranular failures cannot propagate through the view of the map in the *y*-direction.

### 3.4. SCC Cracks

**Figure 8:**
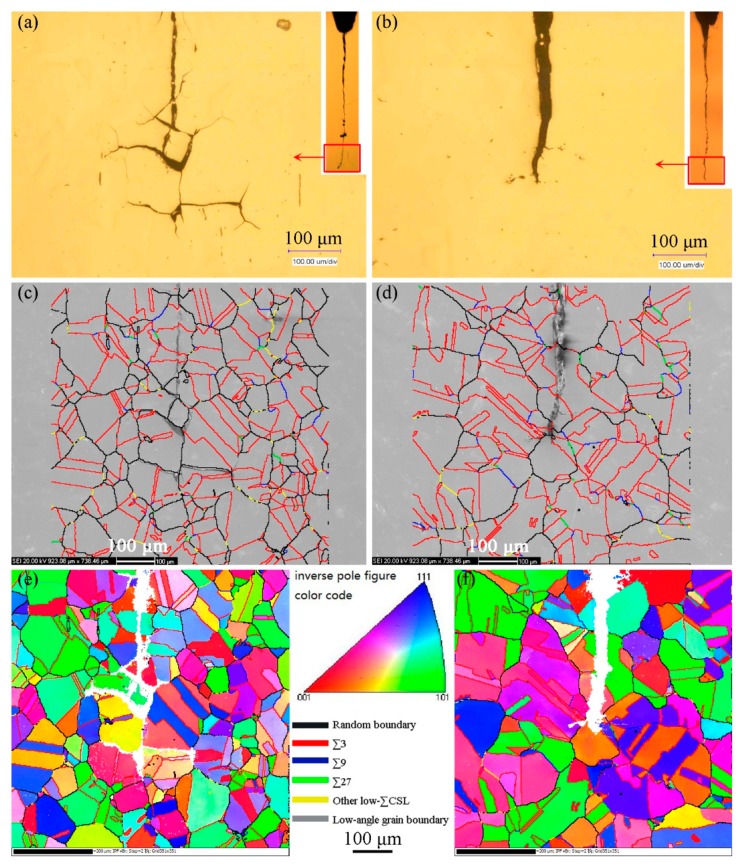
Profile maps of SCC propagation paths of the (**a**,**c**,**e**) non-GBE 316L and the (**b**,**d**,**f**) GBE 316L steel samples. (**a**,**b**) are metallographic maps; (**c**,**d**) are SEM maps overlaid with EBSD-measured GB networks; and (**e**,**f**) are grain orientation maps colored by inverse pole figure (IPF) code.

**Figure 9:**
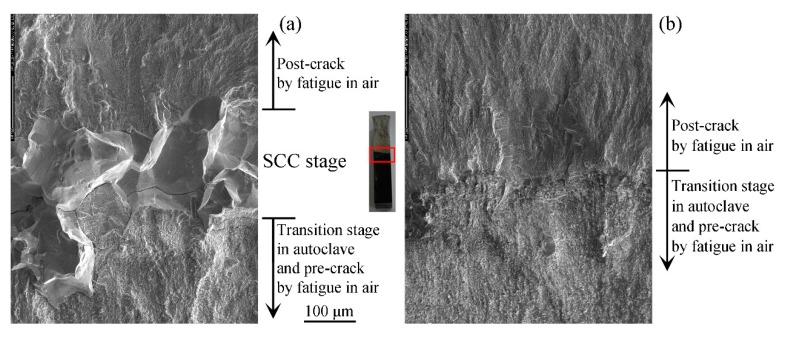
Fracture surface morphologies of the (**a**) non-GBE and (**b**) GBE 316L stainless steel samples after the SCC test.

Comparison tests of SCC on the non-GBE and GBE 316L stainless steel samples in high temperature water were carried out to check the improvement of resistance to intergranular SCC by GBE. The results are shown in Figure 8 and Figure 9. They are observations of the profile surfaces and fracture morphologies at IGSCC propagating regions. The profile maps were observed on the cross-section in the middle of SCC sample thickness. The history of the crack propagation includes three periods: pre-cracking in air, in-situ pre-cracking, and SCC. In the initial period, that is, the pre-cracking with cyclic loading, the crack propagated in the transgranular model whether the samples were processed by GBE or not. Subsequently, the crack propagating model changed from transgranular to predominantly intergranular during the transition and SCC periods. In the SCC period, although the two samples underwent the same load under the same water condition, the behaviors of crack propagation were different. An intergranular crack with a length approximately 200 μm can be observed in the non-GBE 316L, but there is no intergranular crack in the GBE sample, as shown in Figure 9. Even the *K* subjected on the GBE sample is a little larger than that subjected on the non-GBE sample (pre-crack length is a little longer for the GBE sample). This result suggests that the GBE processing gives 316L stainless steel a higher resistance to intergranular SCC.

Figure 8c (non-GBE 316L) demonstrates that the intergranular SCC propagated mainly along random boundaries. The twin boundaries did not fail, although there were a great number of twin boundaries (48.7%). This result is in accordance with past studies [5,7,16,17] which concluded that special boundaries, especially twin boundaries, had superior resistance to intergranular degradation than random boundaries. Figure 8d (GBE 316L) showed that the crack did not grow in the SCC period, although random boundaries existed at the crack front. The higher proportion of twin boundaries (63.7%) is an apparently possible reason. The twin boundaries not only exhibited a strong resistance to cracking themselves, but they also prevented their neighboring boundaries from cracking [20,51]. The cracking of these random boundaries at the crack front in Figure 8d was restrained by the many twin boundaries that were located beside these random boundaries.

## 4. Discussion

### 4.1. Thermo-mechanical Process for GBE

The procedure to obtain a high proportion of low-*∑* CSL boundaries is listed at the top of items involved in GBE [6,13,19,30,33,45,47]. Several Thermo-mechanical procedures were successful in developing the high proportion. These procedures can be classified into two categories: low-strain deformation followed by annealing, and repeated low-strain deformation and annealing treatment for several cycles. Cold-rolling has been widely used to obtain the low-strain deformation for both categories. However, for an industrial application, cold-rolling is not always applicable, especially when processing large-size materials. In this paper, a TMP procedure via warm-rolling was tested for GBE treatment on a large-size 316L stainless steel sample with a thickness of 20 mm. The thickness of the material after GBE processing was 19 mm, so that standard 1/2T CT specimens could be machined from the GBE material. EBSD mapping on the cross-section showed that the 316L stainless steel after the GBE processing had a uniform microstructure with more than 70% of low-*∑* CSL boundaries.

### 4.2. Quantification of the Extent of GBE

It has been well known that low-*∑* CSL boundaries, especially *∑*3 boundaries, have greater resistance to intergranular failure than random boundaries [1,2,3,4,5,7,13,14,15,16,17,45]. This is the basic philosophy for the improvement of material properties by GBE. Therefore, the high proportion of low-*∑* CSL boundaries is one of the main focuses of GBE treatment. For example, in this work, a proportion more than 70% was achieved in the 316L stainless steel after GBE. The SCC test showed that the GBE sample had a higher resistance to intergranular SCC than the conventional (non-GBE) sample of the same material, illustrating the success of the GBE treatment. However, in recent GBE studies, there has been a shift in focus from certain boundary statistics to the topological characteristics of the entire GB network [18]. An interconnected random boundary network constructs the pathway for the propagation of intergranular degradation. The high fraction of low-*∑* CSL boundaries does not necessarily signify interrupting the connectivity of random boundaries. The random boundary connectivity depends not only on the low-*∑* CSL boundary fraction but also on the spatial arrangement of the low-*∑* CSL boundaries in the GB network.

Simulation studies, based on percolation theory, revealed that the threshold number fraction of special boundaries to interrupt the random boundary connectivity of a two-dimensional (2D) network is 0.35 to 0.67 [52]. However, length fractions are more popularly measured in experimental studies. Experimental studies by Michiuchi et al. [45] and Tsurekawa et al. [19] demonstrated that the low-*∑* CSL boundary length fraction of over 70–82% may ensure very low percolation probability of random boundary networks. 3D simulation shows that the threshold number fraction is 0.775 to 0.85 for interrupting the random boundary connectivity in a 3D microstructure [12], which is obviously higher than the proportion calculated by 2D simulation. Experimental studies on 3D GB networks are rare [27] due to the difficulty of 3D characterization. The method required to evaluate the connectivity of a 3D random boundary network is still unclear. In addition, these low-*∑* CSL boundaries are not distributed randomly in the GB network but rather form grain clusters [24,25,26,27], as described in the previous section of this paper and past sources [23,24,25,26]. Connectivity calculation based on percolation theory requires random distribution, suggesting that the simulation studies are not always correct in predicting the connectivity of a real random boundary network. Therefore, it is still difficult to evaluate the random boundary connectivity using the special boundary proportion.

Grain cluster size is another frequently considered parameter when one evaluates GBE microstructures. According to the formation mechanism of grain clusters during GBE processing, GBE microstructures necessarily have connected random boundaries (non-twin boundaries) whether the special boundary proportion is high or low. Therefore, how to quantify the difference of the random boundary networks in the conventional sample and GBE sample, and further evaluate their behaviors during intergranular failure, is a significant undertaking. The connected random boundary network offers pathways for intergranular failure propagation. The number of pathways and the level of difficulty for propagating along the pathway are two parameters for evaluating the random boundary network. There are obviously fewer pathways for the GBE microstructure than for the conventional microstructure. However, how to quantify the difficulty of intergranular failure propagation along a certain pathway is not easy. For a material block with certain dimensions, the length of a pathway through the block should be proportional to the difficulty. A longer pathway indicates that the intergranular failure has to propagate along a more zigzag route [7]. Therefore, a through-view random boundary path (TRBP) was proposed in this work. “Through-view” means the pathway is through the EBSD mapping field of view, supposing that the field of view can represent the entire material block. The normalized length (*D*_R_) of the minimum TRBP for a certain size of EBSD map can represent the difficulty for the intergranular failure propagating through the map.

TRBP was proposed on the basis of grain cluster analysis, and it is a method of GB cluster analysis. Another excellent method for GB cluster analysis is maximum random boundary connectivity (MRBC) that was proposed by Kobayashi et al. [1,53]. MRBC was proposed on the basis of a fractal analysis of the GB network to evaluate the connectivity of random boundaries. The fractal analysis is an excellent idea to quantify the connectivity of a random boundary network. Following the fractal analysis, an examination of several 316L stainless steel samples with different extents of GBE revealed that the fractal dimension of MRBC decreased as the fraction of low-*∑* CSL boundaries increased. The samples with a lower fractal dimension showed a higher resistance to intergranular corrosion.

After comparison, the MRBC and the minimum TRBP have something in common. Both of them represent quantities of measurements of a connected random boundary network. However, the differences between them are apparent. They were proposed on the basis of different opinions about the GB network. Kobayashi et al. [1,53] believed that the random boundary network could be disrupted after GBE processing because all low-*∑* CSL boundaries were believed to be corrosion-resistant boundaries. Only short-range connected random boundaries existed in GBE materials. Thus, the MRBC is a measurement of the maximum connected random boundaries in the view of a measured microstructure map. On the contrary, in the current work, only twin boundaries were believed to be corrosion-resistant boundaries [3,4,5,16,17,53]; therefore, long-range connected corrosion-susceptible boundaries (or non-twin boundaries) existed even in GBE materials, as shown in Figure 6. TRBP focuses on the minimum connected random boundaries through the view of measured microstructure maps.

**Figure 10:**
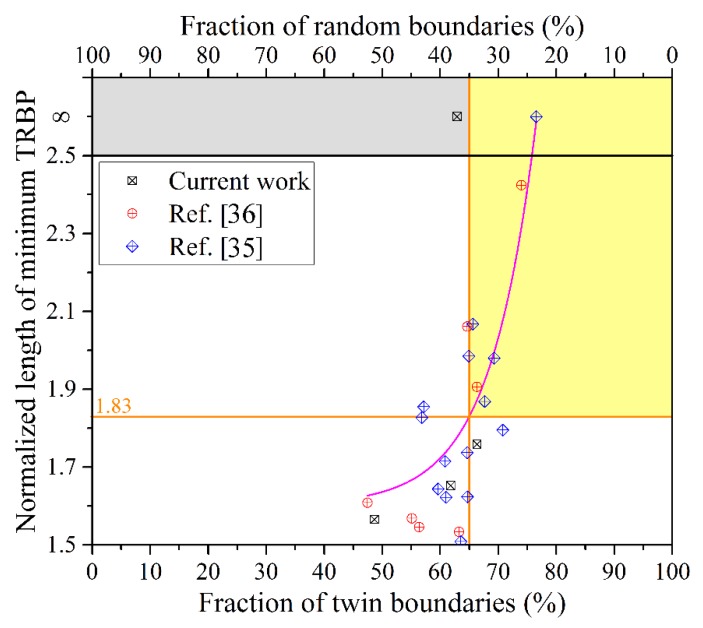
Relationship between the normalized length of the minimum TRBP (*D*_R_) and the fraction of twin boundaries or random boundaries (non-twin boundaries).

Figure 7 shows the minimum TRBPs and twin boundary fractions of the non-GBE and GBE samples. However, a universal relationship between the normalized length of minimum TRBP (*D*_R_) and the fraction of twin boundaries or random (corrosion-susceptible) boundaries is more informative, as shown in Figure 10. Some data published in our previous works [35,36] were cited in Figure 10, noting that only the TRBP in the *x*-direction was calculated and included here. The value of *D*_R_ tends to increase monotonically as the fraction of the twin boundaries increases, particularly when the fraction is more than 65%. This result is in accordance with the percolation threshold value of a random boundary network that was determined by theoretical calculation by Schuh et al. [52] and by experimental statistics by Kobayashi et al. [1]. The *D*_R_ is approximately 1.83 according to the fitting curve of these experimental data when the threshold value of the twin boundary fraction to interrupt the random boundary connectivity was estimated at approximately 65%, as shown in Figure 10. A higher *D*_R_ indicates that the intergranular failure must pass through a longer zigzag path to propagate through a certain distance in a metal block; meanwhile, the zigzag path makes the propagation slower and more difficult [7]. Therefore, materials with a *D*_R_ more than 1.83 and a twin boundary fraction more than 65%, that is, located in the yellow area in Figure 10, must have a much higher resistance to intergranular failure.

## 5. Conclusions

To estimate the improvement of intergranular failure resistance of 316L stainless steel by GBE, the characteristics of a GB network before and after GBE were investigated and compared from the perspective of *∑*3*^n^* boundary fractions, grain clusters and random boundary connectivity. SCC testing of the materials was carried out to prove the efficacy of GBE. The main conclusions drawn from this study are as follows.

(1) Warm-rolling plus annealing is an applicable procedure to increase the fraction of low-*∑* CSL boundaries of large-sized 316L stainless steel in terms of GBE. The SCC test shows that the GBE 316L sample exhibited a higher resistance to intergranular SCC than the sample without GBE treatment.
(2) The high fraction of *∑*3*^n^* boundaries is a highly desired result of GBE processing. However, there is a large difference between the boundary number fraction and the boundary length fraction. Although the length fraction of *∑*3*^n^* boundaries can be increased to more than 70% after GBE, the number fraction is only approximately 50%. This result is correlated with the mechanism difference to form the *∑*3*^n^* boundaries. Most of the *∑*3 boundaries were generated via twinning operations, and they had a larger size on average. All the high-order *∑*3*^n^* boundaries were formed by encounters of grain growth, having a smaller size on average.
(3) A connected non-twin boundary network still exists in the 316L after GBE. The relatively low number fraction of twin boundaries and the formation of grain clusters are contributing factors.
(4) The term through-view random boundary path (TRBP) is proposed to evaluate the extent of GBE. As the twin boundary fraction increases, not only does the number of TRBPs decrease, but the normalized length of the minimum TRBP (*D*_R_) increases monotonically, which leads to intergranular SCC propagating through a longer path with zigzag. Therefore, intergranular SCC becomes more difficult and even prevented.

## Figures and Tables

**Table 1:** Chemical composition of the 316L stainless steel (weight percent, wt %).

Fe	C	Si	Mn	P	S	Cr	Ni	Mo
Balance	0.028	0.47	1.03	0.044	0.005	16.26	10.10	2.08
